# Extremely Low Frequency Magnetic Fields Induce Spermatogenic Germ Cell Apoptosis: Possible Mechanism

**DOI:** 10.1155/2014/567183

**Published:** 2014-06-15

**Authors:** Sang-Kon Lee, Sungman Park, Yoon-Myoung Gimm, Yoon-Won Kim

**Affiliations:** ^1^Department of Urology, Sacred Heart Hospital, School of Medicine, Hallym University, 153 Kyo-dong, Gangwon, Chuncheon 200-704, Republic of Korea; ^2^Institute of Medical Science, School of Medicine, Hallym University, Gangwon, Chuncheon 200-702, Republic of Korea; ^3^School of Electronics and Electrical Engineering, Dankook University, 152 Jukjeon-ro, Suji-gu, Gyeonggi, Yongin 448-701, Republic of Korea; ^4^Department of Microbiology, School of Medicine, Hallym University, Gangwon, Chuncheon 200-702, Republic of Korea

## Abstract

The energy generated by an extremely low frequency electromagnetic field (ELF-EMF) is too weak to directly induce genotoxicity. However, it is reported that an extremely low frequency magnetic field (ELF-MF) is related to DNA strand breakage and apoptosis. The testes that conduct spermatogenesis through a dynamic cellular process involving meiosis and mitosis seem vulnerable to external stress such as heat, MF exposure, and chemical or physical agents. Nevertheless the results regarding adverse effects of ELF-EMF on human or animal reproductive functions are inconclusive. According to the guideline of the International Commission on Non-Ionizing Radiation Protection (ICNIRP; 2010) for limiting exposure to time-varying MF (1 Hz to 100 kHz), overall conclusion of epidemiologic studies has not consistently shown an association between human adverse reproductive outcomes and maternal or paternal exposure to low frequency fields. In animal studies there is no compelling evidence of causal relationship between prenatal development and ELF-MF exposure. However there is increasing evidence that EL-EMF exposure is involved with germ cell apoptosis in testes. Biophysical mechanism by which ELF-MF induces germ cell apoptosis has not been established. This review proposes the possible mechanism of germ cell apoptosis in testes induced by ELF-MF.

## 1. Introduction

Human beings are surrounded by the usage of various electric devices, and the exposure to extremely low frequency electromagnetic field (ELF-EMF) is increasing. It is a growing concern whether EMF induces biological effects which might be harmful to human health. Even though previous research results reported that the genotoxic potential of ELF-EMF has been contradictory, the International Agency for Research on Cancer (IARC) has classified ELF-MF as being “possibly carcinogenic” on the basis of the epidemiologic results on childhood leukemia [1, 2]. Human-made EMF, such as nonionizing radiation, is classified into three categories: extremely low frequency fields (1 Hz–100 Hz), high frequency fields in the band of radio frequency fields (100 kHz–3 GHz), and microwaves (above 3 GHz).

The direct biological effects of an electromagnetic field are divided into thermal effects by electromagnetic field energy absorption, stimulation function by induced electric current, and athermic action by long-term exposure [3, 4]. The mechanisms of biological effects differ according to varying frequency of EMF. Thermal effects mainly occur over 100 kHz radiofrequency. On the other hand, ELF-EMF which is not specially too high does not cause a rise in temperature. It is generally accepted that the energy generated by ELF-EMF is too weak to damage DNA directly leading to genotoxic effects. A recent review of published results including animal studies and* in vitro* studies related to the induction of DNA strand breakage by extremely low frequency magnetic field (ELF-MF) exposure shows conflicting findings [1]. It is suggested that magnetic field (MF) could act as a coinducer of DNA damage rather than as a genotoxic agent per se. Genotoxic effects of EMF may occur indirectly by the generation of oxygen radicals or impairment of radical scavenging mechanism [5]. Proposed biological and biochemical responses of EMF effects are variable, including cell proliferation [6, 7], cell shape, plasma membrane structure modifications [8], alteration of gene expression [9], and apoptosis induction [10]. The International Commission on Non-Ionizing Radiation Protection (ICNIRP) issued a separate guideline for the low frequency range from 1 Hz to 100 kHz to provide protection against adverse health effects [11].

The epidemiology study investigating the reproductive effect of residential exposure to ELF-MF has not found the relationship between MF and reproductive outcome such as fetal loss, pregnancy loss, and miscarriage, or is inconclusive due to the low number of personnel [12–14]. According to the ICNIRP guideline for limiting exposure to time-varying EMF (1 Hz to 100 kHz), overall conclusion of epidemiologic studies has not consistently shown an association between human adverse reproductive outcomes and maternal or paternal exposure to low frequency fields [11].

In the biological effect of ELF-EMF, MF is more important than electric field (EF) because MF induces an electric current in the body but EF does not. The induced electric current on the external surface is considerably greater than that on the internal organs on the basis of dosimetric results from exposure of ELF-MF to the whole human body [11]. Testes superficially located could be more affected by MF than internal organs. Spermatogenesis in testes is a complex process from spermatogonia (2N diploid), primary spermatocyte (2N), secondary spermatocyte (1N haploid), round spermatid (1N), and elongated spermatid (1N) to sperm (1N) through a series of events involving mitosis, meiosis, and cellular differentiation [15, 16]. Hence it could be accepted that testis is one of the most vulnerable organs in the body to external stimuli such as heat, MF, or chemical agents. However the mechanisms involved in the reported adverse effects on reproductive function remain unestablished. Long-term effects of chronic exposure have been excluded from the scope of the ICNIRP's guidelines because of insufficient consistent scientific evidence to fix the thresholds for such possible effects. Nevertheless there is increasing evidence of adverse effects of chronic exposure to ELF-MF on the male reproductive system in animal studies [17–21].

Continuous exposure to a 60 Hz ELF-MF in mice induced apoptosis of spermatogenic cell in duration- and dose-dependent manner [18, 20, 21]. Apoptosis, also called programmed cell death, is a key phenomenon in the control of sperm production. It is suggested that surplus cells and genetically abnormal cells are spontaneously eliminated by apoptosis as a defense mechanism during spermatogenesis [22]. The regulation of germ cell apoptosis during spermatogenesis is mediated by signals derived from the Sertoli cells with which each germ cell is closely associated.

Prominent histopathological alteration in testes exposed to ELF-MF increased germ cell apoptosis and decreased mature spermatogenic cells, especially sperm [17–20]. To date, the reported results regarding the biological effect of ELF-EMF were contradictory due to the variability of exposure system, exposure condition including dose and duration, and material animal including species and age. Therefore, it is not easy to find the casual relationship between ELF-EMF exposure and experimental results. The limited epidemiological and biological data reported, concerning the male reproductive system exposed to ELF-EMF, are comprehensively reviewed in this paper.

**Figure 1:**
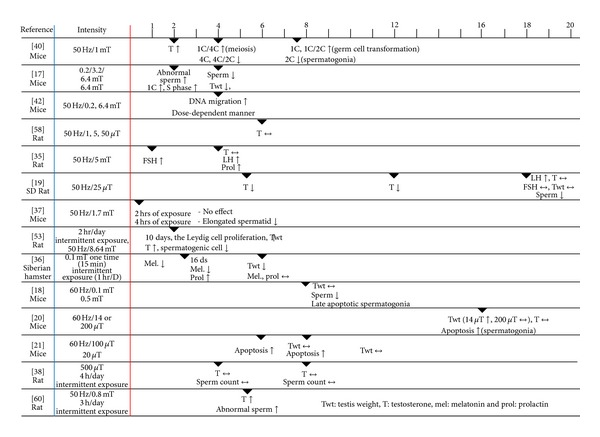
Biological effect of ELF-EMF exposure on male reproductive function in mammal.

The goal of this paper is to infer hypothetical process of germ cell apoptosis on the basis of the serial responses induced by biological effects on reproductive function following ELF-EMF exposure (Figure 1).

## 2. Effects on Reproductive Function and EMF Exposure

Epidemiologic studies on the effects of ELF-EMF on reproductive function have been contradictory since 1986 when it was reported that electric blankets and heated water usage may increase the abortion rate and underweight delivery [23]. However the effects of heat could not link to those of EMF. Two prospective studies showed that no association was found between low birth weight or the rate of spontaneous abortion and use of electric bed heaters [12, 24]. In ICNIRP review, epidemiologic investigations of reproductive health in relation to magnetic field exposure among maternal users of video display terminals have shown no casual relationship [25].

In mammals, prenatal exposure to ELF does not increase miscarriage and gross external, visceral, or skeletal malformations using fields up to 20 mT [26–30]. There is some consistency in increase of minor skeleton alterations in several experiments [26, 27, 31–33]. The skeletal variation may result from statistical fluctuation and is often considered biologically insignificant [34].

Exposure to 50 Hz ELF-MF has effects on sperm parameters in mice, alteration of sex hormones in rat. [17, 19, 35]. Acute 60 Hz magnetic field exposure can result in altered pineal gland and hypothalamic-pituitary-gonadal function in hamster [36]. Continuous exposure to a 60 Hz ELF-MF in mice induced apoptosis of spermatogenic cell in duration- and dose-dependent manner [18, 20, 21]. In quantitative analysis, mature spermatogenic cells (spermatid, spermatozoa) are significantly lower in exposed mice than that in the control [18]. In contrast occupational exposure or short-time exposure to 50 Hz ELF-MF showed no adverse effects on spermatogenesis [37, 38]. There are several studies concerning cell differentiation under EMF exposure conditions [6, 7, 39]. This result demonstrates that cell proliferation and DNA damage induced by ELF-EMF in a specific type of cell may be mediated by an increase of nucleotide mismatch. It is suggested that the exposure to ELF-EMF caused a transient mitogenic effect, followed by a DNA damaging effect [7].

## 3. Apoptosis and MF Exposure

There is continuously increasing evidence of adverse effects of ELF-MF on testes in mammals. The mechanism of germ cell apoptosis induced by exposure to ELF-MF is not well understood. The continuous exposure to a 60 Hz MF at 100 *μ*T for 8 weeks or at 14 *μ*T for 16 weeks induces testicular germ cell apoptosis in mice [18, 20]. More recently, it is found that the apoptosis of testicular germ cell in mice is induced at the minimum dose of 20 *μ*T at continuous exposure to a 60 Hz MF for 8 weeks and the minimum duration is 6 weeks at continuous exposure to 100 *μ*T [21].

In flow cytometry study of mice exposed to ELF-MF of 6.4 mT for 2 weeks, the percentages of round spermatid cells (1N haploid cell) with 1 copy number (23 chromatids in human cell) were significantly lower than that of the control, followed by sperm count lower than those of the control at 4 weeks of exposure [17]. In groups exposed to 50 Hz EMF of 1.7 mT for 4 h, a significant decrease in elongated spermatids was observed at 28 days after treatment [37]. There were no remarkable changes in those of spermatogonia (2N diploid cell) and differentiating spermatocyte (4N tetraploid cell), which is in intervening gap (G2) phase. Moreover, the cell percentage of DNA replication in *S* phase at spermatogonia (chromosome duplication of spermatogonia) increased significantly after 2 weeks of exposure [17]. However in animals exposed for 52 days the cell population in 1N and the 1N : 2N ratio (total germ cell transformation) were significantly higher, and the cell population in 2N spermatogonia was significantly lower than the corresponding control groups [40]. It has been suggested that long-term exposure to an ELF-MF had a possible effect on the proliferation and differentiation of spermatogonia. In mice with 8 weeks of exposure to 60 Hz MF of 0.1 mT or 0.5 mT, flow cytometry study showed the increased late apoptosis of testicular germ cell in the exposed group [18]. Moreover the quantitative analysis by testicular biopsy score showed a significant decrease of mature spermatogenic cell or spermatozoa in the exposed group. It has been accepted that there is a high correlation between testicular biopsy score count and sperm count [41]. In addition, EL-EMF may have the potential to induce directly DNA strand breakage in testicular cell and sperm chromatin condensation in mice [42]. As a consequence, the possible sequential cytotoxic effects induced by MF exposure on testes are a decrease in mature spermatid cells in early phase of EMF exposure followed by proliferation of spermatogonia (increase of germ cell transformation) and degeneration of differentiating spermatogonia in late phase.

Apoptosis, in contrast to necrosis, is morphologically characterized by the condensation of nuclear chromatin at the periphery of nuclei and the fragmentation of the cell following the apoptotic bodies. In the early stage during apoptosis, the chromatin is marginated to the nuclear periphery and is finally completely condensed throughout the nucleus. In the intermediate stage, the cytoplasmic organelles accumulate in an area opposite to the eccentrically located nucleus. In the late stage, disruption of the nuclear remnant is noted, with small areas of condensed chromatin and loss of cytoplasmic organelles [43]. In ELF-EMF exposed mice, terminal deoxynucleotidyl transferase dUTP nick end labeling (TUNEL) positive cell is prominent in spermatogonia [20]. Interestingly, in mouse testes irradiated with single doses of gamma rays of 0.5 Gy, the numbers of TUNEL positive spermatogonia reached a peak of 12 hours after irradiation and then declined. However the typical morphological characteristics of apoptosis, such as margination of chromatin or nuclear fragmentation, were rare [44].

The pathway of apoptosis induced by MF exposure may be different from those induced by the aging process, heat, or hormonal deprivation [45]. The study of exogenous glucocorticoid induced germ cell apoptosis demonstrates that the TUNEL positive cells observed are mostly spermatogonia [46]. In aging men, the main mechanism of germ cell loss is an accelerated apoptosis of primary spermatocytes, whereas the apoptotic rate in spermatogonia is significantly lower [47]. In mice, spontaneous apoptosis is most commonly observed in spermatocytes, including the dividing spermatocytes, less frequently in spermatogonia, and seldom in spermatids [48]. Electron micrographs of apoptotic germ cell in seminiferous tubule from BALB/c mice exposed to 14 *μ*T or 200 *μ*T MF for 16 weeks revealed degeneration of spermatogonia with amorphous electron-dense chromosomal aggregation in the nucleus [20]. The mechanism responsible for spontaneous spermatogonial deletion in rat is not necrosis. Ultrastructural finding of apoptotic spermatogonia is categorized as late stage according to sequential phase of spermatogonial apoptosis [49].

Continuous exposure to a 60 Hz MF may affect biological processes including apoptotic cell death and spermatogenesis in the male reproductive system of mice in duration- and dose-dependent manner [21]. To induce apoptosis of testicular germ cell in mice, a minimum dose is 20 *μ*T at continuous exposure to a 60 Hz MF for 8 weeks, and a minimum duration is 6 weeks at continuous exposure to 100 *μ*T [21]. In mice exposed to 60 Hz MF of 1.7 mT for 4 hours, a significant decrease in elongated spermatids was observed at 28 days after treatment [37].

## 4. Sperm Count and Testis Weight

Testis volume reflects seminiferous tubule involving spermatogenesis. The lumen diameter of the seminiferous tubule may be regulated by elongated spermatid in rats [16]. Even though mature spermatid or epididymal sperm count significantly decreased in ELF-MF exposed animals, there are no significant differences in testis weight [18, 19, 21]. Interestingly testis weight increased in the 14 *μ*T MF for 16 weeks of exposure group compared to that in the sham control group, while it remained unaffected in the 200 *μ*T MF for 8 weeks of exposure group and the 0.1 or 0.5 mT exposure groups [18, 20]. Another study of the same group has shown no association between a decrease in mature spermatogenic cells and alteration of testis weight for 8 weeks of ELF-MF exposure [18]. In another report, the sperm amount decreased after MF exposure for 4 weeks without significant histopathological changes in the mice. Concomitantly the testicular weight was significantly lower than that of the control [17]. Stressful conditions to the testis such as MF exposure or induction of gonadotropin deficiency, which affects the early germ cell development and the reduced spermatogenesis, may not be reflected in reduced sperm counts in the ejaculate until months later.

For long-term exposure of 46 weeks to ELF-MF of 0.1 or 0.5 mT, testis weight decreased in mice of the first and the second generation. The reduction rate on the second generation decreased significantly by about 60%, compared with 10% in the first generation, whereas testis weight was unaffected in the third generation [50]. Histological findings of testis showed that no significant histological changes were observed in that of the first generation, while an increase of phagocytic cells and active spermatogenesis were characterized in that of the second generation but not in the third generation. These results suggest that a decline in testis weight of the second generation is related to histopathological changes. The hypothesis is that long-term continuous exposure may induce adaptive mechanisms, which protect the DNA from harmful influences.

The application of intermittent ELF-MF resulted in a significant increase of DNA damage in contrast to continuous ELF-MF exposure [51]. The intermittent exposure to 50 Hz ELF-MF of 500 *μ*T, which is the European reference level for occupational exposure, had no adverse effects on spermatogenesis applied 4 hours per day for 4 or 8 weeks [38]. In addition there were no significant differences between ELF-MF exposed rat and sham control in measurement of parameter for oxidative stress. It suggests that relatively low intensity and short-term exposure to EMF may have no adverse effect on spermatogenesis [38].

According to the guideline for low frequency MF (1 Hz to 100 kHz) of the ICNIRP, safety levels at short-term exposure are 1 mT for occupational exposure and 200 *μ*T for the general population after 2010 [11]. Those safety levels are double the field density compared to those of the previous guideline. Until now, safety levels for long-term exposure are not determined. The extent of damage would depend on the density of magnetic fields, the duration of exposure, and the time of recovery. At continuous 60 Hz MF exposure, the minimum dose is 20 *μ*T for 8 weeks to induce the apoptosis of testicular germ cell in mice [21], whereas intermittent exposure to ELF-MF, as low as 70 *μ*T, induced genotoxic effects [52].

## 5. Cell Proliferation and EMF Exposure

There are several studies concerning cell differentiation under EMF exposure conditions [6, 7, 39]. Cell proliferation and DNA synthesis are related to initial induction of mutation. In HL-60 leukemia cells, rat-1 fibroblasts, and WI-38 diploid fibroblasts exposed to ELF-EMF, dose-dependent increase was observed in the proliferation rate in all cell types, followed by both increased DNA strand breakage and formation of 8-hydroxy-2′-deoxyguanosine (8-OHdG), one of the predominant forms at lesion of radical-induced DNA damage. The effects of ELF-EMF on cell proliferation and DNA damage were prevented by antioxidant treatment [7].* In vitro* study has shown that human normal osteoblast cell required minimal exposure time to MF exposure to increase cell proliferation [39]. ELF-EMF stimulation for 10 days on undescended testis resulted in the proliferation of the Leydig cell, which is interstitial cell at testis, and an increase in testosterone level and testis weight [53]. ELF-EMF exposure increased the human chorionic gonadotropin- (HCG-) stimulated testosterone producing capacity of the mouse Leydig cells* ex vivo* [54]. Since they may not be hormonally mediated, the possible biological effects of ELF-EMF on alteration of the Leydig cells seem to involve direct cytotoxic effects [55]. In another study, a significant increase in the size and weight of the testicle was due to an increase in interstitial tissue and an elevated level of the testosterone level after a 10-week exposure to 50 Hz of 100 *μ*T MF [56]. We suggest that the Leydig cell proliferation may occur at relatively early phase after MF exposure, accompanied with increase of testosterone level.

In a flow cytometry study, there were no remarkable changes in those of 2C or 4C cells. DNA content in different ploidy cells of the mice exposed to 6.4 mT decreased. Moreover, the cell percentage in *S* phase significantly increased, followed by a decreased sperm count and a decrease in testis weight [17]. In another flow cytometry analysis of animals exposed for 52 days, total germ cell transformation was significantly higher, and the cell population in spermatogonia was significantly lower than the corresponding control groups [40]. This result suggests that long-term exposure to an ELF-MF had a possible effect on the proliferation and differentiation of spermatogonia.

## 6. Hypothalamic Pituitary Gonadal (HPG) Axis and EMF Exposure

Testosterone is crucial for the differentiation of spermatogonia to round or elongated spermatids. Deprivations of gonadotropin or testosterone induce germ cell apoptosis [57]. In rats or mice, exposure to ELF-MF did not affect the serum testosterone level [20, 35, 38, 58]. In exposed rats, follicle-stimulating hormone (FSH) increased in 1 week and luteinizing hormone (LH) increased in 4 weeks after exposure without significant change of testosterone level [35]. FSH level reflects activity of spermatogenesis. The consistent findings are that mature spermatogenic cell, such as spermatid and sperm, decreased in relatively early phase of EMF exposure [17, 53]. In rat, at the tubule stages in which spermatogonia undergo spontaneous apoptosis, FSH response is rising to maximum levels [59].

The hypothesis is that, in the early phase after ELF-MF exposure, a decrease in mature spermatogenic cells, such as round and elongated spermatid, may stimulate FSH secretion in pituitary gland with positive feedback. Thereafter it may be followed by a decrease in testosterone level due to the possibly damaged Leydig cells. And testosterone level is supposed to be partially recovered by upregulation of pituitary gonadotropin. On the other hand, 13 days of short-period exposure to MF in mice showed a rise in testosterone level [40].* In vivo* experiment, EMF stimulation of 2 hours per day for 10 days results in the Leydig cell proliferation, increases in testosterone level and testis weight but decreases in germ cell population [53]. In rat study, ELF-MF exposure of 3 hours per day for 5 weeks in 0.8 mT MF of 50 Hz increased in testosterone level [60]. In* in vitro* study, exposure to 50 Hz of 100 *μ*T for 48 hours markedly increased testosterone production in the mouse Leydig cell cultures. HCG stimulation did not affect testosterone level [55]. These results indicate that an increase in testosterone is not mediated by gonadotropin.

**Figure 2:**
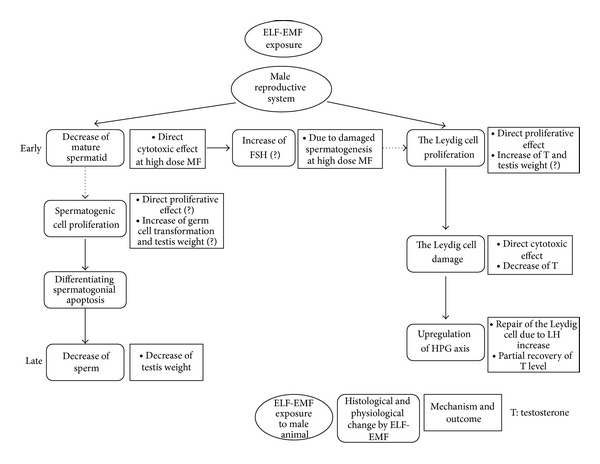
Proposed diagram of apoptotic process in testis exposed to ELF-EMF.

On the other hand, testosterone levels significantly decreased only after 6 and 12 weeks of the exposure period, while the serum levels of LH significantly increased after 18 weeks of exposure concomitant with unaffected testosterone and FSH levels [19]. We speculate that a decline in testosterone level stimulates positive feedback to hypothalamic-pituitary-gonadal (HPG) axis. It seems that testosterone level transiently increases in the early phase of MF exposure due to the Leydig cell proliferation followed by a decline of testosterone production due to the MF induced damaged Leydig cells [35, 53]. An increase in testosterone level in mice exposed to 200 *μ*T ELF-MF for 16 weeks is observed in spite of marked increased germ cell apoptosis [20]. It suggests that biological effect of MF exposure on germ cell apoptosis may not be hormonally mediated. Therefore, cellular proliferation of the Leydig cells may be induced by ELF-MF exposure at relatively early phase. Consequently, testosterone production transiently increased, followed by decreased testosterone production due to damage of the Leydig cells. A decrease in testosterone level may stimulate LH production [19, 35]. The damaged Leydig cells induced by MF exposure may be repaired even though germ cell death occurs.* In vitro* study has shown that ELF-MF influence proliferation and DNA damage in both normal and tumor cells through the action of free radical species [7]. The susceptibility to biological action of ELF-EMF may be different according to the cell type [61]. The Leydig cell may be more resistant to EMF exposure than germ cell (Figure 2).

Acute MF exposure can result in altered pineal gland and HPG function [36]. One-time or intermittent exposure to 60 Hz MF at 0.1 mT was associated with a reduction in melatonin concentration. Daily intermittent exposures for 16 days increased prolactin levels. However, at 42 days, there are no significant changes in melatonin or prolactin levels [36]. It suggests that pituitary gonadal axis may be adapted to chronic exposure to EMF.

## 7. Summary and Conclusion

Germ cell apoptosis can be triggered by hormonal and nonhormonal factors, including gonadal toxin, heat stress, biochemical agents, and EMF exposure. The mechanism of germ cell apoptotic pathway of exposure to ELF-EMF is little understood. However, on the basis of serial biological response induced by ELF-EMF exposure from each of the results reported, we can comprehensively speculate regarding germ cell apoptotic pathway of ELF-MF exposure that initially mature spermatids degenerate due to direct cytotoxicity of high dose EMF. In addition, the production of testosterone transiently increases in the early phase of exposure to ELF-EMF due to the altered proliferation of the Leydig cells. Proliferation rate of spermatogonia and spermatocyte (germ cell transformation) subsequently increases. Proliferation of the Leydig cells is followed by DNA damage. Over time, the testosterone level shows a declining tendency. In the late phase, the Leydig cells get repaired, and accordingly HPG axis is adapted to chronic stimulation of MF. Consequently, the testosterone level partially recovers. On the other hand, germ cell apoptosis results in degeneration of differentiating spermatocyte and spermatogonia. It may be a dynamic compensatory mechanism of spermatogenesis during germ cell apoptosis responding to exposure to ELF-EMF according to intensity of EMF and exposure pattern, age, and duration. To understand the mechanism regulating ELM-MF induced germ cell apoptosis, molecular signaling pathway should be elucidated.
